# Introduction of Ebola virus into a remote border district of Sierra Leone, 2014: use of field epidemiology and RNA sequencing to describe chains of transmission

**DOI:** 10.1017/S0950268819000104

**Published:** 2019-02-22

**Authors:** M. B. DeSilva, T. Styles, C. Basler, F. L. Moses, F. Husain, M. Reichler, S. Whitmer, J. McAuley, E. Belay, M. Friedman, I. S. Muoghalu, P. Swaray, U. Ströher, J. T. Redd

**Affiliations:** 1 Centers for Disease Control and Prevention, Atlanta, Georgia, USA; 2 District Medical Officer-Koinadugu, Ministry of Health and Sanitation, Kabala, Sierra Leone; 3 World Health Organization, Koinadugu, Sierra Leone

**Keywords:** Ebola virus, outbreaks, public health

## Abstract

In early October 2014, 7 months after the 2014–2015 Ebola epidemic in West Africa began, a cluster of reported deaths in Koinadugu, a remote district of Sierra Leone, was the first evidence of Ebola virus disease (Ebola) in the district. Prior to this event, geographic isolation was thought to have prevented the introduction of Ebola to this area. We describe our initial investigation of this cluster of deaths and subsequent public health actions after Ebola was confirmed, and present challenges to our investigation and methods of overcoming them. We present a transmission tree and results of whole genome sequencing of selected isolates to identify the source of infection in Koinadugu and demonstrate transmission between its villages. Koinadugu's experience highlights the danger of assuming that remote location and geographic isolation can prevent the spread of Ebola, but also demonstrates how deployment of rapid field response teams can help limit spread once Ebola is detected.

## Introduction

On 25 May 2014, the first confirmed case of Ebola virus disease (Ebola) was reported in Sierra Leone [1, 2]. As of 20 September 2014 all districts in Sierra Leone except for Koinadugu district had confirmed Ebola cases [3]. Koinadugu was seen at the time as an Ebola success story, the result of early, concerted efforts by the district task force and a prominent private donor who implemented checkpoints for temperature monitoring and a pass system to restrict movement into and out of the district [4]. The district's inaccessibility and limited access points were thought to have helped with this effort.

**Fig. 1.**
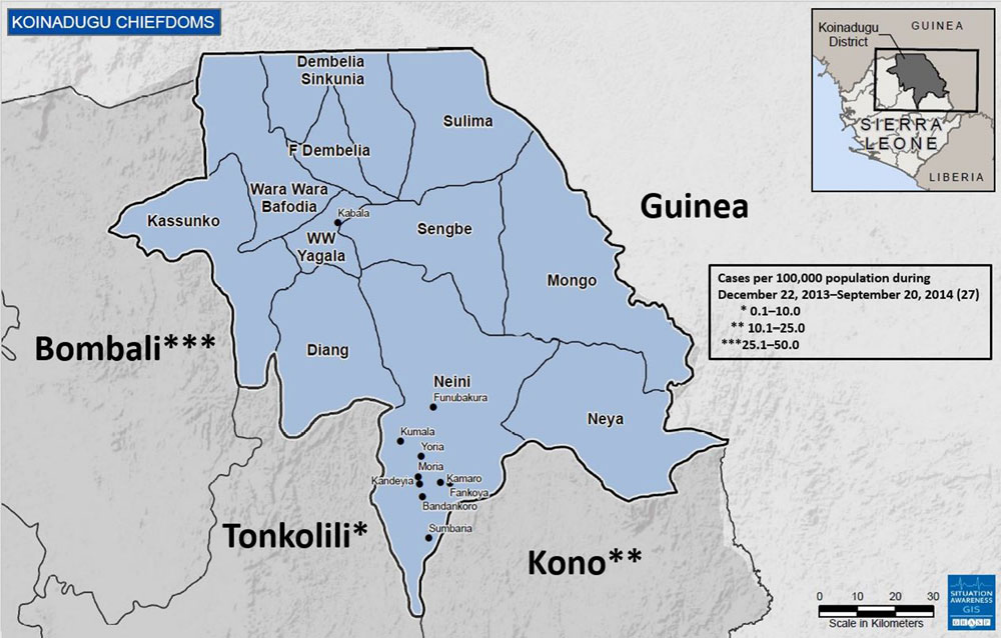
Map of Koinadugu district of Sierra Leone and surrounding Sierra Leone districts and country of Guinea. All Koinadugu chiefdoms are represented as well as nine cities and villages: Kabala, Funubakura, Kumala, Yoria, Moria, Kandeyia, Kamaro, Fankoya, Bandankoro and Sumbaria. The cumulative numbers of cases in the surrounding Sierra Leone districts prior to the first detected Ebola case in Koinadugu are presented – Koinadugu, Sierra Leone, 2014.

Koinadugu, Sierra Leone's northernmost district (Fig. 1), is the largest district geographically with an area of 2121 km^2^ but has a relatively small population of 265 765 based on the 2004 census [5]. Koinadugu is a remote, mountainous district primarily known for farming, and contains 11 chiefdoms (Fig. 1). Nieni chiefdom is the largest geographically, with an estimated population of 39 107 [5]. Nieni has many rural villages, rugged terrain making overland travel difficult, and limited access, including inadequate telecommunications networks.

Koinadugu is bordered by the Republic of Guinea, nearly 300 km from where the Ebola outbreak in West Africa was first identified. Porous borders between the Republic of Guinea and Sierra Leone allow for potential cross-border population movements. The Bombali, Tonkolili and Kono Districts of Sierra Leone make up Koinadugu's other borders (Fig. 1); each reported multiple confirmed Ebola cases during May–October 2014. Koinadugu has one hospital and 70 outlying peripheral health units. During the beginning of the Ebola outbreak in West Africa, the closest Ebola treatment units (ETU) and laboratory facilities for Ebola testing were in Freetown and Bo (Fig. 1). Both of these cities are located approximately 4 h by car from Kabala, Koinadugu's capital, and 8 h or more from Koinadugu's more remote villages. One Ebola isolation holding unit was established in June 2014 to house and care for any suspected Ebola cases in Kabala.

In early October 2014, the Koinadugu District Health Management Team (DHMT) was notified of an unexpected cluster of deaths in Fankoya, a remote village in Nieni chiefdom (Fig. 1). The initial investigation was inconclusive as the deceased were already buried and the village was closed to outsiders. On 11 October, more deaths and illnesses were reported, prompting a public health team to investigate. Two of four specimens obtained on 11 October from deceased persons were positive for Ebola, confirming the presence of Ebola in Koinadugu. This report describes the subsequent outbreak investigation, focusing on how and when Ebola may have been introduced into the district, and reviews strategies for response activities aimed at reducing Ebola transmission. While the West Africa Ebola outbreak primarily affected urban areas, this investigation offered the opportunity to analyse spread to an isolated community from the time of initial detection until control.

## Methods

The work reported in this manuscript represents activities performed as part of a public health emergency response and received human subjects review board waiver from the United States Centers for Disease Control and Prevention (CDC) and the Sierra Leone Ministry of Health.

### Case definitions

Ebola cases were classified as suspected, probable or confirmed using surveillance case definitions issued by the Sierra Leone Ministry of Health and Sanitation (MoHS) [6]. Suspected and probable Ebola cases were identified by calls from concerned community members or healthcare workers to the DHMT (aka, ‘alert calls’), contact tracing or upon presentation for clinical care. Local health teams completed case investigation forms (CIFs) for persons under investigation for Ebola to collect demographic data, exposure to potential contacts 21 days prior to symptom onset and funeral attendance. Clinical specimens were collected for Ebola virus testing when possible. Clinical outcome data and burial information were reviewed, when available. CIF data were entered into the Epi-Info™ Viral Hemorrhagic Fever database (VHF) and updated with case outcome information and laboratory data. Confirmed cases had a positive laboratory test for Ebola virus (EBOV) by reverse transcription polymerase chain reaction (RT-PCR).

### Ebola virus testing

Blood specimens were obtained from living suspected and probable cases using collection protocols developed by MoHS. Oral swabs were collected from deceased persons prior to burial. In situations where blood collection on living persons was not possible, oral swabs were accepted for testing, but were not recommended based on a lower sensitivity [7, 8]. Diagnostic tests for Ebola virus RNA were performed by quantitative RT-PCR assay of blood or oral swabs at laboratories in Sierra Leone. RT-PCR results for suspected and probable cases were considered negative if test results were negative for specimens collected between 3 and 10 days after illness onset. Laboratory results and case identification number were recorded in an Excel spreadsheet and transferred to the corresponding records in the VHF.

### Fankoya investigation

After molecular testing of samples obtained on 11 October 2014 confirmed Ebola in Koinadugu, DHMT staff and a five-member joint WHO–CDC public health team travelled to Fankoya during 16–18 October 2014 and conducted verbal autopsies on the initial cluster of deaths in Fankoya from community members. No CIFs were completed for these deaths, but during semi-standardised interviews demographic information including name, age, sex, village of residence, burial information and potential contact with other Ebola cases was collected when possible. In Sierra Leone, one of the local customs necessary for a robust response is gaining the support of local leaders [9]. Paramount Chiefs represent the highest authority in a Chiefdom; without the Paramount Chief's support, communities may refuse to cooperate with outside partners, and delays in the initiation of response activities may occur. Thus, it was necessary to gain the Paramount Chief's permission in order to perform ongoing outbreak response.

### Transmission tree

We constructed a transmission tree based on demographic information, laboratory results and disease outcome to understand outbreak propagation in Nieni chiefdom and to estimate the mean number of secondary cases by generation of disease transmission. Deaths identified during the initial investigation in Fankoya were included in the transmission tree if both demographic and illness information were available. We also used the transmission tree to identify possible ‘super-spreaders’ – a term we applied to individuals that appeared to spread EBOV disproportionately more than other infected individuals [10].

### Genome sequencing

Genomic sequencing of eight specimens was performed retrospectively at CDC, after control of the outbreak, to establish the geographic origin of Nieni's cluster as well as determine the degree of connectedness between cases. Samples were selected based on Ebola virus threshold cycle (Ct) value and sample volume. Blood samples were inactivated under Biosafety level 4 biocontainment (CDC, Atlanta, GA). Nucleic acid was extracted using a MagMAX Pathogen RNA/DNA isolation kit (Invitrogen); DNA, ribosomal and carrier RNA were removed and unbiased deep sequencing was performed according to methods used by Matranga *et al*. [11]. Consensus genomes were generated using the IRMA-Ebola pipeline [12]. The nucleotide substitution model (GTR + Γ (*n* = 4)) was chosen using JModelTest, version 2.0 and maximum likelihood phylogenetic trees were constructed using phyml, version 3.0 with approximately 1500 full-length EBOV genomes; median joining networks [13] were constructed using PopArt with a subset of full length viral sequences. Branch support estimates were generated using the most conservative value returned from *χ*^2^ parametric and SH-like non-parametric approximate likelihood ratio test (phyml argument –b −3). New genomes acquired from clinical specimens were deposited into GenBank: MG948587-91.

### Statistical analyses

We used SAS version 9.3 (SAS Institute Inc., USA) to link VHF and laboratory databases. We report results of descriptive analyses as frequencies, proportions, means and medians. Statistical analysis of continuous variables was performed using the Student's *t*-test to compare means with the assumption of unequal variance; differences in categorical variables were assessed using Fisher's exact test. We calculate the odds ratio for differences in completed CIFs based on the time period during the outbreak.

## Results

### Fankoya field investigation

The initial investigation in Fankoya revealed 24 deaths, of which 22 met the suspected or probable case definition for Ebola, and five additional living persons ill with Ebola-like symptoms. Demographic information, illness onset and date of death were obtained for 11 (50%) of 22 deaths; investigators were unable to locate surrogates for the remaining 11. Samples were taken from four (80%) of five actively symptomatic patients; three of which were confirmed positive for Ebola virus by RT-PCR. Of 11 deaths for whom information was collected, surrogates provided anecdotal information about funeral attendance (often in nearby villages) or direct contact with a deceased person. Funeral attendance was traced as far back as early September, nearly 6 weeks prior to the first confirmed Ebola case in the district. Based on information collected during these interviews, the index case in Koinadugu was believed to be a business traveller from a neighbouring district whose family lived in Fankoya. After further interviews, it was determined that the earliest case in Fankoya was a young male who returned home after visiting Kono district.

### Intensified outbreak response

After confirming the first Ebola cases in Nieni Chiefdom, Nieni's Paramount Chief gave permission for public health and medical teams to be stationed in Nieni to provide active case- and contact-finding, specimen collection, basic medical care for suspected Ebola patients, community engagement and training about safe burials practices. Surveillance, laboratory, ambulance and burial teams from Kabala were assigned to 1-week rotations in Nieni starting 29 October.

WHO established a temporary community care centre (CCC) in Kumala on 30 October while community members modified an existing school building to function as a longer-term CCC. The school-based CCC began operations on 12 November. To expedite laboratory testing, CDC, the United Nations (UN) and other partners arranged for thrice-weekly helicopter transport of laboratory specimens from Koinadugu to CDCs field laboratory in Bo District starting the week of 24–29 November.

### Case finding efforts and response improvement

After Ebola was reported in Nieni, surveillance teams and contact tracers were mobilised and additional staff were trained to increase response efforts. Improved case finding meant that CIFs were completed for all living suspected and probable cases identified after 20 October. During 19 October–1 November, the mean number of days from symptom onset to case report completion for patients meeting the epidemiologic case definitions was 7.3 days after excluding two outliers; the mean was shorter for confirmed cases with 6.8 days. During 2–15 November, after addition of more response team staff, the mean number of days decreased to 4.3 days (difference in means = 3 days (95% confidence interval (CI) 1.2–4.8 days)) for all case definitions and to 4.4 days for confirmed cases (difference in means = 2.4 days (95% CI 0.3–4.4 days)). The percent of confirmed cases for whom CIFs were completed while living increased from 74% (17/23) to 94% (38/41) during these same time periods, though not statistically significant (odds ratio 4.5, 95% CI 1.0–20.0).

### Ebola virus testing

**Fig. 2.**
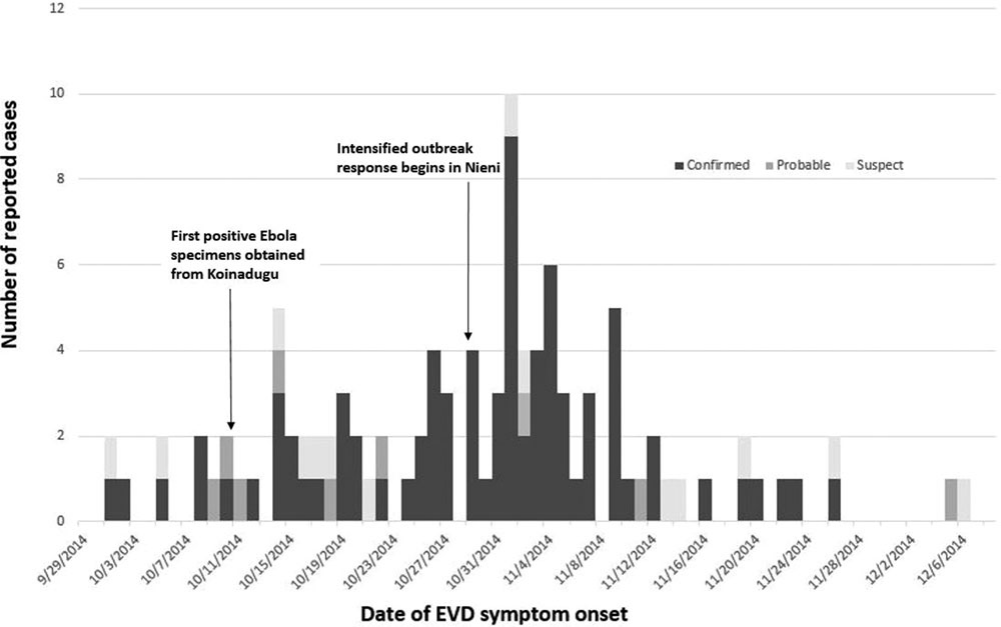
Number of reported Ebola cases in Koinadugu district, Sierra Leone, reported by week of symptom onset – Sierra Leone, 2014.

After the first confirmed case was reported in early October, 203 suspected and probable Ebola cases from Koinadugu were tested for Ebola virus RNA from 129 blood specimens and 74 oral swabs. Quantitative RT-PCR testing of 129 whole blood specimens revealed 67 (52%) confirmed cases of Ebola, 58 (45%) non-cases and four (3%) undetermined cases. Cases were considered undetermined if samples lacked required information such as collection date or there was a need for a repeat sample due to collection of the first sample within the first 72 h of illness onset. Quantitative RT-PCR testing of oral swabs identified 22 (30%) confirmed Ebola cases, 49 (66%) non-cases and three (4%) undetermined cases. Combining this laboratory information with CIF records resulted in 89 confirmed Ebola patients, 14 probable cases and 18 suspected cases during 1 October–17 December (Fig. 2). Due to lack of recording vital status at the time of oral swab collection, we were unable to determine how many cases were dead at the time of collection. The median age for confirmed cases was 34 years (IQR 22–47.5). Confirmed cases were predominantly female (53/89; 60%).

### Transmission tree

**Fig. 3.**
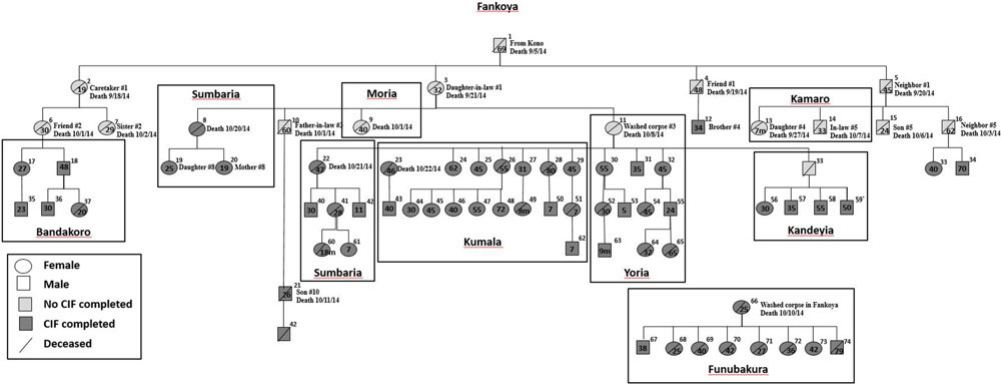
Ebola transmission tree for Nieni Chiefdom, Koinadugu district, Sierra Leone, 2014. Squares represent males and circles represent females. The age of cases in years unless otherwise noted, is provided within the circle or square if available. The village of where the person became ill or was buried is Fankoya unless noted by a box around a case or group of cases with a different village listed: Bandakoro, Sumbaria, Kandeyia, Moria, Sumbaria, Kumala, Yoria, Kamaro or Funubakura. Information about epidemiologic connections between the first 25 cases and case number 66 are listed if available.

We constructed a transmission tree (Fig. 3) during November which included 11 of the 22 deaths thought to be related to Ebola found during the initial investigation in Fankoya. By 9 December, the transmission tree was expanded to a total of 74 suspected or confirmed Ebola cases including 38 (51.4%) deaths. Included cases were predominantly female (60.8%), had a median age of 35.5 years (range 0.6–80 years), and were from 10 villages. The most common exposures reported were direct person-to-person contact (86.5%; 64/74) or participation in burial practices (32.4%; 24/74). Ten persons indicated both exposures. Four persons denied both contact with sick patients and funeral attendance.

The number of secondary cases per generation of disease for the first, second, third and fourth generations was 4, 11, 18 and 24, respectively. This leads to a rough empirical estimate of the reproduction number according to disease generation decreasing from four in the first generation of disease to approximately 1.3 in the fourth generation. The transmission tree demonstrates spread of Ebola from village to village within Nieni chiefdom. Starting in Fankoya, Ebola was transmitted to residents of nine other villages in Koinadugu (Fig. 3). Case investigations identified three possible super-spreaders (Fig. 3, case ID#s 11, 26 and 66); mean number of secondary cases per super-spreader was 8.3 cases (range 5–12).

### Genome sequencing

**Fig. 4.**
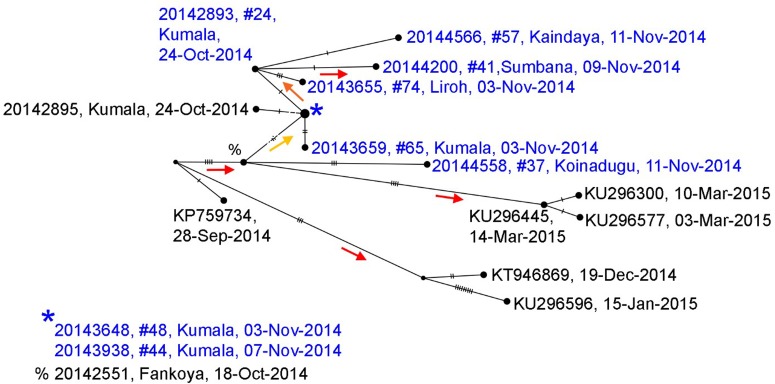
Median joining haplotype network based on full genome sequences generated from samples collected in Nieni Chiefdom, Koinadugu district, Sierra Leone, 2014. Viral sequences were selected from the Nieni-specific clade and closely related clades from a maximum likelihood tree constructed with all available Ebola sequences (Supplementary Figs. 1 and 2). Nodes are placed according to sample collection dates and scaled to represent number of identical sequences. Individuals included in the transmission tree (Fig. 4) are highlighted in blue. Nodes without labels represent hypothetical ancestors and vertical hash marks represent number of nucleotide changes. Statistical support (aLRT from maximum likelihood trees) for nodes is indicated by coloured arrows which point to supported nodes: red >0.9, orange >0.8, yellow >0.75.

Whole genome sequences of Ebola collected during the Koinadugu investigation and processed after control of the outbreak, are more closely related to sequences from Sierra Leone than sequences from Guinea (Supplementary Fig. 1). The phylogenetic tree (Supplementary Fig. 2) shows strong support for the sequences identified from Nieni clustering together on the same clade – this clade includes samples that were collected from the same geographic region and time, but sequenced at different laboratories (Fig. 4) [14]. The earliest collected sample associated with the Nieni clade was collected from Fankoya (%, 20 142 551) and is consistent with the transmission tree illustrating that the initial source of infection in the Nieni chiefdom was from Fankoya. The phylogenetic tree supports a relationship for transmission between Kumala (cases #24, 44, 48 and 65) and Sumbaria (case #41), Kandeyia (case #57) and Funubakura (case #74). The case on the phylogenetic tree from Bandakoro (case #37), shares the common Fankoya case, but is separate from the Kumala-related cases represented, consistent with the transmission tree (Figs. 3–4).

### Deaths and burial information

Early investigations in Fankoya revealed suspected Ebola deaths in early September. However, due to limited resources of burial and surveillance teams, CIFs were not routinely collected for deceased persons until 20 October. During 5 September through 17 December, a total of 56 potentially Ebola-related deaths were reported in Nieni including 16 suspected, eight probable and 32 confirmed Ebola cases. Burial information was missing for 26 deaths (which occurred before CIFs were routinely collected); it is assumed these burials were performed in the community by friends and family members. For 30 deaths for whom burial information was recorded, 26 (87%) were buried safely by a trained burial team and four (13%) were buried by the community using traditional practices. The majority of these deaths took place after intensification of staff and other resources.

## Discussion

Investigation of Ebola in Koinadugu, a particularly remote area, determined that Ebola was likely to have been introduced to Koinadugu weeks to months prior to confirmation of Ebola in the district. Whole genome sequencing results suggest the initial route of Ebola transmission into Koinadugu was from within Sierra Leone rather than a cross-border transmission. However, based on inferred phylogenetic relationships, Koinadugu likely had multiple introductions of Ebola during the course of the Ebola outbreak. Of note, whole genome analysis demonstrates that while closure of international borders was associated with decreased transmission, international cross-border transmission still occurred [15]. Our investigation also highlights that transmission had already occurred prior to implementation of public health Ebola control efforts, highlighting the danger of accepting lack of case reports as evidence of a lack of disease transmission. Ebola transmission was eventually decreased through a series of proven interventions aimed at early diagnosis, prompt and effective isolation of Ebola patients and implementation of safe burial practices [1, 16, 17].

Access issues, resource limitations and logistical constraints of remote villages are a challenge to outbreak responses and can lead to delayed identification of Ebola-related morbidity and mortality with increased potential for spread to other communities. These challenges have been identified in previous haemorrhagic fever outbreaks, and intervention strategies to overcome these challenges have been noted by previous articles. Information collected about the decrease in number of days from symptom onset to case report completion and the increase in the percent of confirmed case patients for whom CIFs were completed shows how intensive surveillance and enhanced response efforts may improve key responses. During the West Africa Ebola outbreak, which primarily affected urban areas, the challenges of rural environments in Liberia similar to Koinadugu were noted to interfere with Ebola epidemic control [18–20]. In response to these challenges, Liberia implemented a national strategy to rapidly respond to rural outbreaks of Ebola which decreased the duration of rural Ebola outbreaks and associated case-fatality rates [19].

Activities undertaken in Nieni to control the Ebola outbreak were similar to the objectives of Liberia's national strategy. After confirming Ebola cases in Nieni Chiefdom, the DHMT and international partners attempted to increase case finding, improve isolation measures, decrease time to receipt of laboratory results and expedite transfer of confirmed Ebola cases to ETUs. It should be noted that prior to establishment of field-based, rotating surveillance and laboratory teams in Nieni and the Kumala CCC, no confirmed cases from Nieni survived long enough to be transported to an ETU. After these measures were put in place the majority of newly identified suspected patients survived long enough for transport to an ETU, and several patients returned to their villages after ETU discharge. Our data also showed an increase in case reporting and decrease in reporting delays after intensified outbreak response efforts were started.

We determined by verbal autopsy that several cycles of unusual illnesses and deaths which were likely to have been Ebola had occurred approximately 6 weeks before the first reported Ebola case in this district. These findings emphasise the importance of assessing surveillance in silent areas in order to promptly detect ongoing transmission and rapidly implement response measures. Surveillance gaps in Sierra Leone resulting in Ebola-infected persons coming to the attention of health authorities only after they died were also reported by Crowe *et al*. [21]. In response to these gaps, Crowe describes the development of the Community Event-Based Surveillance (CEBS) system, a supplement to the country's existing surveillance system. Sierra Leone has continued use of CEBS (now referred to as Community Based Surveillance, or CBS) as a component of its strategy of Integrated Disease Surveillance and Response (IDSR). This surveillance strategy includes syndromic case definitions for suspected cases of conditions such as VHFs. Its case definitions are appropriate for use in locations such as Sierra Leone and other resource-limited settings.

In addition to Ebola containment and infection prevention strategies, there was a targeted community effort to raise Ebola awareness and decrease stigma related to the disease. The support of Nieni's Paramount Chief of the outbreak response was integral to the successful containment of Ebola. In remote areas where Ebola response activities are limited, integration of existing political and public health structures into response efforts might improve community enthusiasm for participation in the response [22].

Similar to earlier Ebola outbreaks and other areas affected by the Ebola outbreak in West Africa, burial preparation, funeral practices and travel to neighbouring villages contributed significantly to the spread of Ebola in Nieni chiefdom [23, 24]. Whole genome sequencing results reinforce epidemiologic links by mapping cases from different locations and generations of disease to common ancestors. Richards *et al*. describe the social networking of rural communities in Sierra Leone including the complexities and importance of familial lineages and traditional practices of marriage and funerals with regards to the current Ebola outbreak [25]. Villages are linked, sometimes across great distances, through marriage. The fact that only four case patients included in the transmission tree denied both contact with sick patients and funeral attendance suggests that spread of Ebola among villages in Nieni was likely related to travel to care for or attend funerals of sick friends and family. After alerting communities that funeral practices were related to Ebola spread, the percentage of burials conducted by specially trained burial teams increased. However, traditional burials continued. Social mobilisation and community acceptance are necessary for the efforts to change burial practices to be effective.

Limitations of our investigation include incomplete case reporting, particularly at the beginning of the outbreak. Although the initial investigation teams uncovered 22 suspicious deaths, time, logistical and political constraints did not allow for an exhaustive evaluation. When combined with initial delays in identifying cases, a significant spike in case reports followed community notification about the first confirmed case. We believe a more gradual progression of Ebola onsets likely occurred than suggested by the epidemic curve. Similarly, focused interventions in Nieni chiefdom resulted in improved case-finding and were not necessarily emblematic of increased transmission in the area.

## Conclusion

The experience in Koinadugu highlights the danger of assuming that remote location and geographic isolation can prevent the spread of Ebola, and further, that absence of case reports reflects the absence of transmission. People remain mobile and social networks drive the spread of diseases like Ebola regardless of geopolitical boundaries.

Nieni chiefdom's case study underscores the importance of active surveillance for early detection of Ebola and is a call for increased preparedness of rural populations. The ability to (1) set up surveillance using standard case definitions and prompt reporting and (2) quickly enact outbreak control strategies in remote areas is critical for early containment of Ebola [26]. A policy of active surveillance for Ebola virus among all deaths and non-malaria febrile illnesses had not been instituted in Sierra Leone at the time of this outbreak, but was instituted in early 2015. Whether such an active surveillance system would have resulted in an earlier identified outbreak in Nieni and a smaller eventual outbreak size is not clear. The fact that this outbreak was limited to 89 confirmed cases and 14 probable cases speaks highly of the rapid and seemingly effective outbreak response efforts in this remote part of Sierra Leone.

## Supplementary material

For supplementary material accompanying this paper visit https://doi.org/10.1017/S0950268819000104. click here to view supplementary material

## References

[1] Dixon MG and Schafer IJ (2014) Ebola viral disease outbreak – West Africa, 2014. Morbidity and Mortality Weekly Report (MMWR) 63, 548–551. 24964881 PMC5779383

[2] Gire SK (2014) Genomic surveillance elucidates Ebola virus origin and transmission during the 2014 outbreak. Science 345, 1369–1372. 25214632 10.1126/science.1259657 PMC4431643

[3] Incident Management System Ebola Epidemiology Team, CDC, Ministries of Health of Guinea, Sierra Leone, Liberia, Nigeria, and Senegal, Viral Special Pathogens Branch, National Center for Emerging and Zoonotic Infectious Diseases (2014) Ebola virus disease outbreak – West Africa, September 2014. Morbidity and Mortality Weekly Report *(*MMWR*)* 63, 865–866. 25275331 PMC4584876

[4] Frankel TC (2014-) The fight to save the last Ebola-free district in Sierra Leone. *The Washington Post, Retrieved from https://www.washingtonpost.com*.

[5] Statistics Sierra Leone (2006) 2004 Population and Housing Census. Retrieved from http://www.sierra-leone.org/Census/ssl_final_results.pdf.

[6] Dietz PM (2015) Epidemiology and risk factors for Ebola virus disease in Sierra Leone-23 May 2014 to 31 January 2015. Clinical Infectious Diseases 61, 1648–1654. 26179011 10.1093/cid/civ568

[7] Erickson BR (2016) Ebola virus disease diagnostics, Sierra Leone: analysis of real-time reverse transcription-polymerase chain reaction values for clinical blood and oral swab specimens. The Journal of Infectious Diseases 214(suppl 3), S258–S262. 27587631 10.1093/infdis/jiw296 PMC5769963

[8] Grolla A (2011) The use of a mobile laboratory unit in support of patient management and epidemiological surveillance during the 2005 Marburg outbreak in Angola. PLoS Neglected Tropical Diseases 5, e1183. 21629730 10.1371/journal.pntd.0001183 PMC3101190

[9] Roddy P (2007) The Medecins sans frontieres intervention in the Marburg hemorrhagic fever epidemic, Uige, Angola, 2005. II. lessons learned in the community. The Journal of Infectious Diseases 196(Suppl 2), S162–S167. 17940945 10.1086/520544

[10] Galvani AP and May RM (2005) Epidemiology: dimensions of superspreading. Nature 438, 293–295. 16292292 10.1038/438293a PMC7095140

[11] Matranga CB (2014) Enhanced methods for unbiased deep sequencing of Lassa and Ebola RNA viruses from clinical and biological samples. Genome Biology 15, 519. 25403361 10.1186/s13059-014-0519-7 PMC4262991

[12] Shepard SS (2016) Viral deep sequencing needs an adaptive approach: IRMA, the iterative refinement meta-assembler. BMC Genomics 17, 708. 27595578 10.1186/s12864-016-3030-6 PMC5011931

[13] Bandelt HJ, Forster P and Rohl A (1999) Median-joining networks for inferring intraspecific phylogenies. Molecular Biology and Evolution 16, 37–48. 10331250 10.1093/oxfordjournals.molbev.a026036

[14] Park DJ (2015) Ebola virus epidemiology, transmission, and evolution during seven months in Sierra Leone. Cell 161, 1516–1526. 26091036 10.1016/j.cell.2015.06.007 PMC4503805

[15] Dudas G (2017) Virus genomes reveal factors that spread and sustained the Ebola epidemic. Nature 544, 309–315. Epub 2017 Apr 12. 28405027 10.1038/nature22040 PMC5712493

[16] Frieden TR (2014) Ebola 2014–new challenges, new global response and responsibility. New England Journal of Medicine 371, 1177–1180. 25140858 10.1056/NEJMp1409903

[17] Dhillon RS, Srikrishna D and Sachs J (2014) Controlling Ebola: next steps. The Lancet 384, 1409–1411. 10.1016/S0140-6736(14)61696-2 25308287

[18] Summers A (2014) Challenges in responding to the Ebola epidemic – four rural counties, Liberia, August–November 2014. Morbidity and Mortality Weekly Report (MMWR) 63, 1202–1204. 25522089 PMC5779531

[19] Kateh F (2015) Rapid response to Ebola outbreaks in remote areas – Liberia, July–November 2014. Morbidity and Mortality Weekly Report (MMWR) 64, 188–192. 25719682 PMC5779593

[20] Blackley DJ (2015) Rapid intervention to reduce Ebola transmission in a remote village – Gbarpolu County, Liberia, 2014. Morbidity and Mortality Weekly Report (MMWR) 64, 175–178. 25719678 PMC5779598

[21] Crowe S (2015) A plan for community event-based surveillance to reduce Ebola transmission – Sierra Leone, 2014–2015. Morbidity and Mortality Weekly Report (MMWR) 64, 70–73. 25632956 PMC4584562

[22] Hagan JE (2015) Implementation of Ebola case-finding using a village chieftaincy taskforce in a remote outbreak – Liberia, 2014. Morbidity and Mortality Weekly Report (MMWR) 64, 183–185. 25719680 PMC5779599

[23] Centers for Disease Control and Prevention (2001) Outbreak of Ebola hemorrhagic fever Uganda, August 2000–January 2001. Morbidity and Mortality Weekly Report (MMWR) 50, 73–77. 11686289

[24] Chippaux JP (2014) Outbreaks of Ebola virus disease in Africa: the beginnings of a tragic saga. Journal of Venomous Animals and Toxins Including Tropical Diseases 20, 44. 25320574 10.1186/1678-9199-20-44 PMC4197285

[25] Richards P, Amara J, Ferme MC (2015) Social pathways for Ebola Virus Disease in rural Sierra Leone, and some implications for containment. PLOS Neglected Tropical Diseases 9, e0003567. doi: 10.1371/journal.pntd.0003567. PMC4401769 25886400

[26] Okware SI (2002) An outbreak of Ebola in Uganda. Tropical Medicine & International Health 7, 1068–1075. 12460399 10.1046/j.1365-3156.2002.00944.x

