# Supplementary figures and images for: Introduction of Ebola virus into a remote border district of Sierra Leone, 2014: use of field epidemiology and RNA sequencing to describe chains of transmission
Source: Epidemiol Infect. 2019 Feb 22;147:e88. doi: 10.1017/S0950268819000104 (PMC6518841; doi:10.1017/S0950268819000104)

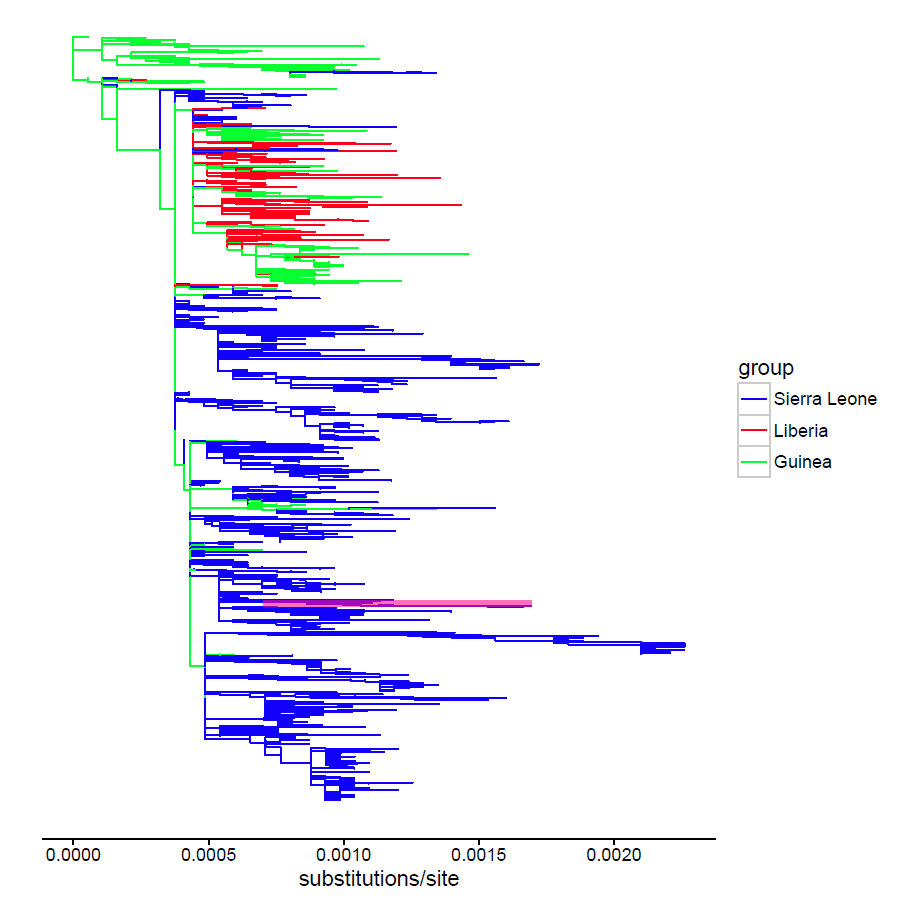

Supplement: Supplementary file 1 [file S0950268819000104sup.zip › S0950268819000104sup001.png]

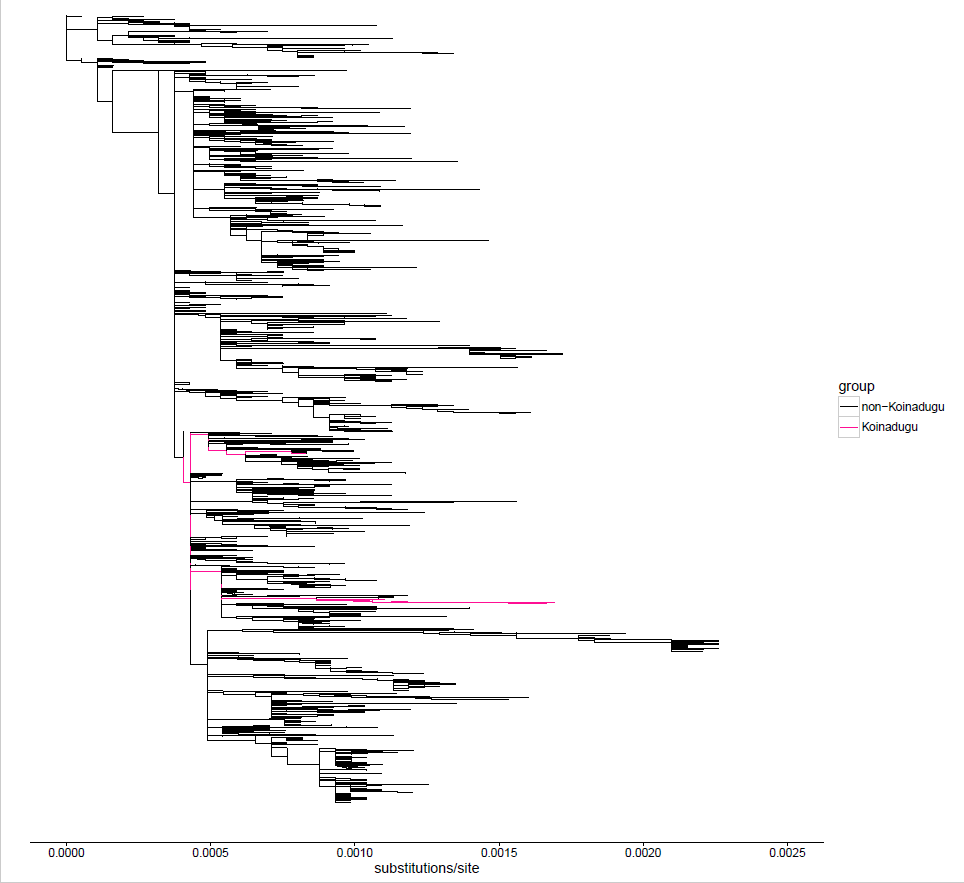

Supplement: Supplementary file 1 [file S0950268819000104sup.zip › S0950268819000104sup002.png]
